# Fatigue and Fear with Shifting Polio Eradication Strategies in India: A Study of Social Resistance to Vaccination

**DOI:** 10.1371/journal.pone.0046274

**Published:** 2012-09-26

**Authors:** Rashid S. Hussain, Stephen T. McGarvey, Tabassam Shahab, Lina M. Fruzzetti

**Affiliations:** 1 Warren Alpert Medical School of Brown University, Providence, Rhode Island, United States of America; 2 Brown University, Department of Epidemiology, International Health Institute, Providence, Rhode Island, United States of America; 3 Jawaharlal Nehru Medical College, Department of Paediatrics, Aligarh, Uttar Pradesh, India; 4 Department of Anthropology, Brown University, Providence, Rhode Island, United States of America; Aga Khan University, Pakistan

## Abstract

Shifting polio eradication strategies may have generated fear and “resistance” to the eradication program in Aligarh, India during the summer of 2009. Participant observation and formal interviews with 107 people from May to August 2009 indicated that the intensified frequency of vaccination was correlated with patients' doubt in the efficacy of the vaccine. This doubt was exacerbated in a few cases as families were uninformed of the use of monovalent mOPV1, while P3 cases continued to occur. Many families had also come to believe that their children had been adversely affected by OPV after being told the vaccine carried no risk. Though polio is now largely eradicated in India, with only a single case in 2011, greater transparency about changes with vaccination policy may need to be considered to build trust with the public in future eradication programs.

## Introduction

As the polio program nears completion in India, with only a single case in 2011, another disease may be eradicated. However, the road to this goal had been made difficult due to social “resistance” when families refused to vaccinate their children. Though “resistance” to the program due to rumours about the vaccine, frustration with the slow pace of development, and fatigue with repeated doses have been documented [1], [2], [3], [4], this qualitative study contextualizes some sources of fear and “resistance” to the program during the summer months of 2009 to provide insight for future eradication endeavours.

During the time of the study, monthly administration of the vaccine without an explanation contributed to families growing tired of the program [1], [2], [3], [4], [5]. Since about 2000, the polio eradication program shifted from vaccinating only a few times a year on National and Sub-National Immunization Days (NIDs) to vaccinating door-to-door every month until children reach the age of six in high-risk areas [5]. Though the door-to-door vaccinations were technically for families who failed to vaccinate their children at established booths, since fewer and fewer families bothered coming to the polio booth, the monthly door-to-door policy had effectively become the standard. This policy shift was also subtly reflected in the UNICEF ad campaign which changed from advocating “do boond zindagi ke liye” or “give two drops for life” to “do boond har baar” or “two drops every time.”

From 2007 through the course of this study in 2009, the Global Polio Eradication Initiative (GPEI) vaccinated almost exclusively with the monovalent oral polio vaccine (mOPV1) in a final push to eradicate P1 [6]. There are three strains of the wild polio virus: P1, P2, and P3, of which P2 has been eradicated using the trivalent oral polio vaccine (OPV) [7]. However, because several studies revealed poor trivalent OPV efficacy for the remaining strains in endemic states, monovalent vaccines were developed [8], [9], [10]. Though a bivalent vaccine targeting both P1 and P3 is now in use [11], eradication strategy at the time of the study focused on P1, resulting in the spread of P3. At the end of 2008, there were 484 cases of P3 and 75 cases of P1 in India, and by the end of 2009 there were 662 P3 cases and 80 P1 [12]. Whereas the strategy to target P1 was probably sound at a biological level, the lack of clear explanation about the use of monovalent vaccines, combined with the confusion due to repetitive rounds, may have contributed to “resistance.”

Possible adverse events associated with OPV may also have contributed to “resistance.” Though the OPV is relatively safe vaccine, as a live-attenuated vaccine, it does have official contraindications including pregnancy or immunodeficiency [13], and carries a small risk of Vaccine-Associated Paralytic Polio (VAPP). Though VAPP is rare, occurring approximately in one out of every one million children vaccinated, it is clinically identical to paralytic poliomyelitis [7]. Additionally, in India is has been found that the risk may be elevated to 1 per 143 000 infants born [14], possibly due to the increased coverage, though this has not been confirmed [15].

Thus, this study was conducted to identify social factors associated with “resistance” to the polio eradication program. The study focused initially on “resistance” regarding rumours of sterilization amongst the Muslim community which will be explained in a separate paper. When it became apparent that “resistance” had spread into the non-Muslim community, the study was broadened to identify knowledge and opinions about the eradication strategy amongst the general public.

## Methods

### Ethics Statement

Proper informed consent was taken for all interviews and financial compensation provided, in accordance with a protocol approved by the Brown University IRB on March 17, 2009. Both verbal and written consent was obtained from literate respondents who were provided a copy of the consent documents for their records. Only verbal consent was obtained if subjects were not literate, though they were provided a copy of a separate consent document for their records. All consent forms and procedures were approved by the Brown University IRB.

### General Protocol

This research took place in the city of Aligarh in Uttar Pradesh, India. Aligarh District was classified as a “high-risk” district for the spread of polio by the GPEI [16] and was the source of the majority of India's polio cases in 2003 [17].

The methods were conducted in accordance with the principles of rapid ethnography/rapid assessment procedures (RAP) and included in-depth key informant interviews, behavioural observation, and semi-focus groups [18].The qualitative data was collected over four months, May–August 2009. Methods included participant observation of two GPEI-sponsored clinics and three week-long door-to-door polio rounds, interviews with 27 stakeholders in the polio program, and 80 semi-structured interviews with families who interacted with the polio program. Informal interviews were embedded within the participant observation while the structured interviews were conducted afterward.

### Participant Observation and Unstructured Interviews

The researcher used the method of participant observation to collect data both at clinics run by the GPEI and the door-to-door vaccination program. During the door-to-door program, the researcher was embedded with polio vaccination teams as they attempted vaccinating families who had actively refused in the past to accept vaccination, described as “resistant” families. Three polio rounds were conducted during the course of the study, preceded by booth days which started on May 26, July 5, and August 9 of 2009. During the rounds, the researcher was perceived to be a part of the vaccination team, and held onto charts and paperwork while observing interactions between the vaccinators and the families. Field notes were taken of families' reaction to the polio program, health conditions in the neighbourhoods, and the behaviour of the information, education and communication (IEC) teams of the SMNet which consisted of UNICEF Community and Block Mobilizing Coordinators (CMCs and BMCs) as well as medical interns from the Jawaharlal Nehru Medical College and Ajmal Khan Tibbiya College. Participant-observation of the GPEI-run clinics included noting interactions between the patients and staff, and conducting 15 informal interviews with clinicians. The participant observation of the polio rounds was used to gain a stronger understanding of the realities of vaccination on the ground, brainstorm challenges to the program, and compare the local situation to that expected from the initial literature review to fit within the theoretical framework of ‘structural violence’ and health justice.

Participant observation at the GPEI paediatric clinic was conducted to provide insight into local health care and needs, while observation of the door to door Pulse Polio rounds provided insight into both workings of the program and families perception of it. A total of 22 informal interviews were also conducted with the vaccination teams during the course of data collection, and transcribed alongside the field notes. The participant observation was also used to generate further research questions, and determine proper sites for the bulk of interview recruitment which occurred afterward. The participant observation stage of the study was not used for active participant recruitment itself.

### Active Recruitment and Structured Interviews

Twenty-seven formal interviews were conducted after active participant recruitment with both grassroots and administrative stakeholders in the polio eradication program. The in-depth structured interviews were conducted with health promoters called Community Mobilizing Coordinators (CMCs) (5), polio booth-workers (5), clinicians who worked in underserved areas (4), medical interns (5), community physicians (5), and administrators with the GPEI (3). These diverse ‘stake holder’ views were conducted to get an insight into how resistance was viewed by those involved with the eradication effort at various levels, and how they saw or sought to address the shifting nature of resistance.

Interview questions were based on data collected from the initial participant observation, and included questions about individuals' knowledge and opinions about the polio eradication program, the oral polio vaccine, causes of “resistance” to vaccination, their confidence in the program's ability to succeed, and their opinions about current or alternative eradication strategies.

Eighty formal, semi-structured interviews were also conducted with families with children who interacted with the polio eradication program in major parts of Aligarh after active recruitment. Individuals from each family were interviewed based on their willingness to participate. All respondents were either the head or co-head of the family as mothers and fathers participated about equally. Though interview questions were geared towards one interviewee, if other family members contributed to the discussion, the interview was allowed to take its course as a semi-focus group. Other than one day of interviews where five “resistant” families were specifically sought out for interview in Jeevangarh, families with children were selected randomly. Participants were recruited by knocking on doors in the major streets/alleys of each ward as determined by neighbourhood informants, asking for families with children who would be willing to interview until a total of around five families were interviewed in each ward as demarcated by local GPEI partner organizations. This occurred in all wards except Jeevangarh which had a total of nine interviewees, four random, and five exclusively “resistant” as described. This partially random selection yielded a diverse number of participants, including several who were “resistant” to vaccination. The number of interviews in each of the wards was Maulana Azad Nagar (5), Jamalpur (5), Civil Lines (3), Jeevangarh (9), Begambagh (5), Devatray (5), K.R. Jain(5), Gandhi Nagar (5), Upper Kot/Upper Fort (5), Bhojpura (6), Shahjamal (6), Indira Nagar (5), Bannadevi (5), PPC (5), and Mehfooz Nagar (6).

These wards represented major blocks of population divided along socioeconomic and religious lines. This sampling was thus sought to get a broad overview of opinions in the community about the polio program, with “resistant” views being well represented. This was why “resistant” individuals were initially sought, though “resistance” was found to be common enough that neighbourhood opportunity sampling yielded resistant families who had interacted with the program. Of all who gave their informed consent to participate in the study, 77 families continued the semi-structured interviews to completion. A total of 3 families from Bhojpura, Jeevangarh, and Mehfooz Nagar decided to stop the interview midway for an unspecified reason. Participants were asked questions about their knowledge and opinions about the polio eradication program, the oral polio vaccine, causes of “resistance” to vaccination, their confidence in the program's ability to succeed, and the provision of health services. Two translators who were familiar with the local environment, fluent in local dialects of Hindustani, and trained to conduct health promotion field activities joined the researcher in conducting interviews. All interviews were conducted in either Hindustani or English by the researcher with the assistance of the translators.

In total, 107 structured or semi-structured interviews were conducted. Participants included 80 families with children in the aforementioned parts of Aligarh, and 27 stakeholders in the eradication program. Each structured interview took 30–40 minutes, and was conducted at a site of the interviewee's choosing. Transcripts were either recorded by hand or with an electronic recorder with the permission of the interviewee. All interviews were made confidential unless the right was specifically waived. Interviews were conducted to the point of saturation, as data repetition occurred at all levels, indicating the views found reflected that of a substantial portion of the studied respondents [19].

### Data Analysis

Analysis of the data was conducted by the researcher independently. All transcription and translation was done by the researcher who is fluent in Hindustani. Due to difficulties with sound quality and local dialects, full transcription was conducted for 20 of the recorded interviews. Partial transcription was done for the remaining 82 recordings in addition to the partial transcription already done in the form of field notes during all structured and semi-structured interviews. Of the structured and semi-structured interviews, 5 were transcribed by hand exclusively. All informal interviews were written alongside field notes. No software was used in the analysis of the data, which was manually coded for causes for “resistance” in the Muslim community, causes for rising “resistance” in the non-Muslim community, gaps in knowledge about polio eradication strategy, behaviour of the polio vaccination teams, and trust of the medical establishment and government. This coding scheme was developed based on previous literature about causes of “resistance” and inductions from the participant observation. The informal interviews from the field notes and transcripts from the interviews were coded and grouped by the described major themes to give a better understanding of “resistance.” Participant observation, semi-structured interviews, and formal interviews based on active recruitment were given equal weight and not differentiated during analysis.

## Results

### Fatigue and Confusion from Program Intensification

During the course of the study, families in Aligarh showed fatigue from vaccinating their children monthly because they did not understand the need to do so. Though most respondents supported the eradication program and vaccinated their children, many did not seem informed why the program had intensified the frequency of vaccination. Families described that when they asked the door-to-door vaccination teams why they visited them so often, they were usually not given an adequate response. Though the presence of medical interns helped, members of the vaccination team were observed sometimes providing dubious etiological explanations to the families: telling them that polio was “special” and needed a constant boost which other vaccines did not. One clinician who worked with routine immunization services explained that many patients did not understand why, whereas they vaccinated with BCG at fifteen days, DPT at one and a half months, and received a measles injection twice, they had to vaccinate for polio almost twelve times a year until they were about six years old. A respondent from a Christian family in Banna Devi explained that she did not understand the shift, or its purpose. Explaining her frustration and confusion about the frequency of the visits, she said:

It (the polio vaccine) is working. But nowadays they are just overdoing it. Coming all the time and bothering people. Like they are coming in the afternoon, which is just a nuisance. They are also overdosing everyone…before it used to be every month. Now it is every week right?!…This has become too much then right?

Though her reference to the vaccination happening every week was likely due to confusion with the “B-team” that vaccinated children missed during the first rounds, it seemed no one informed her why there was a shift in policy.

### Doubts due to Translucent Monovalent Vaccine Policy

Confusion about intensified vaccination was confounded by a lack of information about the strategy to eradicate the P1 strain of the virus through the monovalent mOPV1 vaccine. As families saw polio cases occur despite the intensified rounds, they started to doubt the program. Families were not usually informed of differences between the strains: P1 and P3; and when they saw or heard of polio cases occurring, usually P3 at the time, many came to doubt the efficacy of the monthly administered vaccine, which only targeted P1. Expressing her doubts about the vaccine and frustration with the repeated doses, a Hindu lady in Begambagh explained:

People might think there is no point to the program and refuse to vaccinate on that ground. We think there is no point which is why we don't vaccinate. For the rest, we cannot say. Son, they are giving so much of the vaccine, so much of the vaccine, but still polio is affecting children.

Somewhat informed about the strains, but still confused about the strategy, an educated man in Banna Devi asked:

It is a P3 virus for polio right? It comes up in the newspaper that, despite all the vaccinations, P3 cases are occurring.

He was not informed that the P1 strain was being targeted for vaccination, and was growing tired of the program.

The lack of awareness about the vaccine was often reinforced by the vaccination teams. When providing families with mOPV1 on the polio rounds, vaccinators usually told families that the vaccine protected them from “polio,” and not just the P1 strain. Based on the advice of the vaccinators, families gave their children the vaccine thinking it protected them from “polio” when in fact it protected them only from P1.

If families thought the mOPV1 protected them generically from “polio” it would have proven problematic if P3 cases occurred. Though this likely did not occur amongst selected participants, a Muslim family interviewed in Shahjamal shared a qualitatively similar experience. When family members were asked what they thought of the polio eradication program, the parents responded that they thought the government was trying “to make a fool of the public.” They said that despite vaccinating their son regularly, he became crippled in a manner characteristic of polio. The father said he did not believe in the rumours that the government wanted to sterilize their children, but rather, it was their personal hardship which caused them to lose faith in the program. The full story of the family is outlined below:

Translator: He is saying that the program is making a fool of the public.

Respondent: A fool out of them.

Researcher: Meaning?

Respondent: Making a fool out of them meaning they don't like it. To make crazy…The thing you are trying to say is in front of me…look.

Translator: He is saying to say that this child has polio.

Researcher: He has polio?

Translator: And that he has regularly been drinking the vaccine.

Respondent: And he's been drinking regularly. He's been drinking the vaccine till he was seven years old.

Researcher: Okay okay. And still he got sick?

Respondent: Still he got sick…‥

Researcher: In your view, does this vaccine do any work or what does it do?

Respondent: In our view…they tell us to come and take the vaccine, take the vaccine. Are we not taking the vaccine? No we are not. This is the benefit from the vaccine (pats his crippled child on the back); this is the benefit from it. (Emotionally) This is the benefit from it! Nothing! We shouldn't vaccinate.

Though this man said he once believed in the polio eradication program and vaccinated his children regularly, he had lost faith that the vaccine worked because despite vaccinating, his son became crippled. If the public is not informed of which type of vaccine they receive while cases continue to occur, they may stop supporting health programs.

### Fear from Adverse Events Proximate to Vaccination

Though many people had come to doubt the efficacy of the vaccine, as rumours spread about adverse reactions with the polio vaccine, some individuals also became sceptical of its safety. In fact, one family had become so afraid of the vaccine that on the polio rounds, they threatened to kill their own children, call the police, and frame the polio workers if they did not leave their homes. Throughout Aligarh, there were rumours that when some families vaccinated their children against polio, the next day the children contracted a fever, became afflicted with polio, or even died. In Upper Kot, one grandmother screamed that the vaccine gave one of her grandchildren polio and refused to vaccinate the other grandchildren “even if the Prime Minister of India” came to her door. This “resistance” frustrated the interns who, referring to Vaccine-Associated Paralytic Polio (VAPP), remarked “there is one case in a million and that one case causes so many problems.” Often, rumours indicated that adverse effects occurred when the children were vaccinated during a fever, which is not an official contraindication to OPV vaccination. When vaccinating on the polio rounds, CMCs and medical interns tried to counsel patients about this fact, but many remained fearful. One family in Shahjamal explained to us how they refused to vaccinate because they had heard this rumour:

Respondent's Wife: Okay, so if you drink the vaccine when you are sick it can be problematic right? I have heard it has caused problems. Like when a child is getting a fever and they force you to drink the vaccine…

Respondent's Wife: When we used to take the vaccine, many children had problems because of it. Some children even died.

Respondent: Some children even died!

Researcher: Some even died because of the polio vaccine? Okay okay.

Respondent: When they had a fever.

Respondent's Wife: With Typhoid…sometimes they get the child to drink the vaccine during typhoid fever too…and then it is problematic.

Researcher: So people force you to take the vaccine?

Respondent: The vaccine is given by force.

Respondent's Wife: When we refuse, (the polio workers) say that the vaccine won't cause any harm and give the vaccine. Then the health of the child gets compromised. Quite a few cases like this have happened. That is why a lot of people are afraid.

Thus, the family refused to vaccinate because they heard the polio vaccine caused severe adverse effects during a fever. The fact that vaccinators told them the vaccine wouldn't cause any harm before administering what was a lethal vaccine in the rumour caused families to further distrust the program. Even an individual who was himself tragically afflicted with polio, and thus continued to vaccinate his children, expressed some fears because of this risk:

Researcher: Do you feel the vaccine is safe?

Respondent: I feel it is safe but I have heard of two cases which happened in Jeevangarh. I heard that the children had fevers, but that the polio workers forced their way and vaccinated the children, causing harm to the children. There people had forced their way and were very rude.

Researcher: So this happens?

Respondent: Yes, yes it happens. If you are going to vaccinate a child, you should know everything that is going on (with the child) at first.

Many people were afraid to vaccinate their children because they feared that the polio vaccine actually caused adverse affects such as fevers, diarrhoea, and even paralysis.

However, this fear of adverse reactions was not limited to the polio vaccine alone. For example, when a routine immunization camp funded by the GPEI to increase vaccine acceptance was held in Jeevangarh on June 11, 2009, one of the staff workers informed the visiting physicians and vaccinators that they should not push people to vaccinate. Apparently a child had died in the past few days from an adverse reaction to the DPT vaccination, causing widespread fear and refusal to vaccinate.

### Distrust of the Vaccination Teams

In addition to fears about the vaccine itself, several families expressed distrust of the vaccination teams. Whether educated or not, many doubted the training of the CMCs and BMCs, and felt that both the instances of polio cases occurring despite vaccination and the stories about adverse effects occurred when vaccinators failed to maintain the proper temperature of the vaccine (cold-chain). One educated Hindu resident of Banna Devi explained that he saw polio cases arising despite repeated rounds. He felt the workers might be to blame, breaking the cold-chain:

Respondent: I don't understand how, despite taking the vaccine from our physicians, and taking these door to door vaccines, symptoms (cases) keep arising….This means something is wrong. Either there is something wrong with the medicine, or the proper temperature which the medicine has to be kept at is not maintained. Either somebody is not looking at the expiration date or the workers are being careless in how they maintain the temperature and handle the icebox.

Translator: The cold-chain is broken.

Respondent: Or they hold it in their hands.

Many people, especially from the upper classes, felt that they would not only put their children at risk of adverse events from the broken cold-chain, but possibly other diseases as the teams vaccinated multiple children with the same dropper. Many of these respondents were not afraid of the vaccine per se and said that they would gladly go to a physician or clinic for the same vaccination. One “resistant” family in Upper Kot explained their position as such:

Respondent: No. We don't trust the workers or whoever comes. If we have to take it at the Medical College we will from the doctors but it does not feel right to take it from the polio workers. They are given fifty rupees to wander around and give drops. They might finish a drop and just throw it, and will use the same mouth piece for everyone.

Researcher: So you will vaccinate there but not here?

Respondent: Yes, we will go, show our children to the doctor at the Medical College get routine care and vaccinate and come back.

Researcher: So you don't vaccinate from the workers, you don't like the workers?

Respondent: The workers aren't educated and are just paid fifty rupees to wander; we don't know what they are vaccinating with, what they are giving.

When a businessman from Jamalpur waited at one of the health clinics for medication, looking at the polio vaccinator, he commented on her training and the danger he felt it posed to children:

She has been holding the vial the whole time, warming it. The people do not handle the drops properly; it should be kept in the ice box. For example, when giving the drops, she might put the dropper into a child's mouth and then use the same dropper with the next child, causing contamination.

### Attitudes regarding Policy Transparency

Vaccination teams did not share information with families regarding either the intensification of the vaccine program, use of mOPV1 versus the trivalent OPV, or risks associated with vaccination. Though the rationale for the intensification of the program was simply poorly publicized, officials with the GPEI in Aligarh indicated that it was formal policy to not actively inform the public of other issues discussed in this paper.

Local GPEI officials described that the monovalent strategy to vaccinate against P1 was based on the national strategy, but there no obligation was felt to inform the public of shifts in the type of vaccine provided. As seemed apparent from respondents and observations on the polio rounds, GPEI officials confirmed that the public was not informed that only the monovalent was provided unless they asked though it was, “not as if (they were) hiding the fact either.” However, given that the vast majority of the population was uneducated, unless informed the vaccine protects them from one strain, they would not have been able to ask or find out. As one GPEI official explained:

Look, it is like this, the government has emphasized we should eradicate P1 first, then P3 which is easy. P3 is less virulent, spreads slower, and its residual paralysis effects are weaker. But today we are talking about the community. To the community, these things aren't 100% shared, through the newspapers and other communications we don't always say which vaccine is being used. But if someone asks, it is not as if we are hiding the fact either. Whether paediatricians, private practitioner, or a common man. If someone asks us, we answer and tell them what the strategy is, why P1 is used, why P3 is not, these things aren't hidden on any level, but you can't share it in every community because there are very few people who will understand you.

Responses from key stakeholders regarding sharing risks associated with vaccination, like VAPP, were similar. One of the local community physicians who worked regularly with the polio eradication initiative explained that because people were uneducated, only minor risks associated with vaccination were shared with patients. Major ones like VAPP were ignored because of the risk of rumours. He explained:

They (patients) know about some reactions, but they don't know about serious reactions like paralysis. Nobody talks about paralysis with them, because if we tell them there might be one case, the person will run away, so we generally avoid it. But for minor reactions like development of swelling, that people share.

Researcher: So in general there is the impression is that it is best not to inform then right now?

Respondent: No, we can only inform them if they are literate. Without education, you tell one person, they will tell 100 persons. That will happen.

As seemed apparent from respondents and the polio rounds, GPEI officials confirmed that the public was not informed of the risks associated with vaccination. Instead, vaccinators informed them there was no risk or side effect. As explained by an individual with one GPEI partner organization:

Look, you can explain everything to educated people and they understand everything. But if you talk about VAPP with uneducated or less educated people, about Vaccine Associated Paralytic Polio, it would be taken negatively. For this reason, these technical issues, we do not discuss with them, we just tell them that there is no harm from the vaccine itself, there is no side-effect, and your child will be fully safe.

Paediatricians and physicians who generally worked with the population directly rather than in a more administrative/public health position, were more discouraged by the lack of transparency regarding VAPP. One paediatrician openly complained, “VAPP is kept a secret, people are not told about it, and it remains a rarely discussed issue, even in the medical community.”

## Limitations

Due to the ethnographic approach to the study, in addition to the manual coding of the transcribed data and field notes, this study is subject to researcher's bias. Selection bias may have occurred due to the relatively large yet unspecified number of participants who declined to interview. Additionally, as not all interviews were completely transcribed, the full scope of views shared by participants may not have been acknowledged. Difficulties translating local dialects and poor sound quality may also hinder analysis of the data.. Nevertheless, the study provides insight into views and attitudes toward vaccination which were prevalent at the time of the study.

## Discussion

Given that polio eradication necessitates almost complete vaccination coverage [5], unclear communication about vaccination policy seems to have been problematic amongst study participants. Increased transparency and an adverse-effects compensation program may need to be considered to build more trust with the public in future programs.

Intensification of the polio program and lack of transparency about the use of monovalent vaccine seemed to contribute to “resistance” to the program. Families in cities like Aligarh had not been given adequate explanations as to why the polio eradication program was vaccinating every child every month. From the data, it is apparent that this may have contributed to fatigue, if not suspicion of the program. Because the public was uninformed of the strategy to eradicate P1 first as well as differences between P1 and P3, when P3 cases occurred, many saw a “polio case” generically and came to doubt the efficacy of OPV. Simultaneously, the dearth of this information deprived families of the choice to vaccinate against P3, potentially breaching trust between the patient and provider, as qualitatively was the case with the family in Shahjamal whose child developed polio-like conditions despite vaccinating regularly. These families deserved to know what medications they were, or were not being able to provide for their children.

During the course of this study, it was also found that there were rampant rumours that the OPV caused children to develop fevers, sickness, AFP, or even die. There are three possible causes for these beliefs: that they were coincidental, that they were cases of VAPP, or that they were cases of P3. Most officials insisted that the rumours of fevers, paralysis, and death were coincidental: that the conditions existed at the same time as the administration of the OPV or lay dormant in the children before the administration of the vaccine. Because the vaccine was administered monthly, coincidences are likely in a population where death and disease remain, all too common.

However, the fact that the vaccination teams usually told them the vaccine was *completely* safe made these individuals further doubt the program. This explanation to the public is problematic, if not dishonest. With all vaccines, there is some inherent amount of risk. The OPV, as a live attenuated vaccine, carries the risk of causing either a fever, mild body aches, or even full fledged paralysis if the virus reverts [20]. As these were the very conditions patients described their children had after taking the vaccine, it would beg to ask the question if cases of Vaccine Associated Polio (both with and without paralysis) were occurring.

Though GPEI officials in Aligarh insisted that not a single case of VAPP occurred in the district since the start of the program, VAPP cases have occurred in India [21], [22]. It has been estimated that there may have been from 83 to as many as 300 cases of VAPP per year in India during the course of the program [4], [23]. There were also 21 cases of Vaccine Derived Polio Viruses (VDPV) in India during the course of 2009 [12]. A third possible cause for these rumours might have been occurrences of P3 which happened proximate mOPV1 vaccination. Regardless of whether the cases described in this paper were coincidental, cases of VAPP, VDPV, or P3, their effects represent how adverse affects like VAPP cause public apprehension and distrust when full information is not disclosed.

Das and Das have described trust as fundamental for effective immunization, for even with poor information, people rely on the trust with their provider to accept the vaccine [24], [25]. Though rumours about vaccine failure and the arguments presented for “resistance” are often deemed “unsound from a biomedical perspective,” they are often based on rational arguments and have a strong emotional aspect due to their personal nature [26]. Studies in risk assessment have demonstrated that biased media coverage, and anxiety-provoking incidents, as was the case here, cause uncertainties to be denied and risk perception to be exaggerated [27]. Thus, though the statistics for such cases are small, the emotional impact of each incident is large for the family of an affected child, and has similar reverberations when the story is spread, forming “shared notions of resistance” [28]. As stories spread of adverse events proximate to vaccination it was this trust which was shaken, causing an increase in “resistance.”

Though the policies of not disclosing the risks associated with OPV vaccination or explaining the monovalent strategy were initially done to avoid confusion and achieve high levels of vaccination, if trust with the public was affected, it would have been important to increase policy transparency and improve information, education, and communication (IEC) activities.

Risk perception studies indicate that the public “will accept risk from voluntary activities that are roughly 1000 times as great as it would tolerate involuntary risks,” highlighting the importance to increase active demand for the vaccine [27]. The Ottawa and Bangkok charters for Health Promotion advocate for increases in health literacy as a means for improving public control over all modifiable determinants of health [29]. With increased health literacy, communities are often better able to determine what is best for their well-being, and advocate for programs like vaccination. For example, Friedman and Shepeard's study on HPV vaccine attitudes in the US found that though initially many participants did not know about HPV or risks associated with the vaccine, when empowered and informed, participants clarified their concerns and actively demanded the vaccine. As valued members, the participants also input their own ideas about how to best inform rather than alarm the public about the disease which was highly sensitive issue due to its high prevalence, nature as an STI, and carcinogenicity [30]. For example, some African Americans in the group recommended supplying the vaccine through private clinics with African American physicians rather than government health agencies due to the historical legacy of government distrust from the Tuskegee study, drawing parallels with “resistance” to OPV due to historically based distrust of the government amongst the Muslim community in India.

Most notably, increases in IEC activities have already improved delivery of vaccine with polio eradication in India. Mobilization of grassroots CMCs and BMCs to increase interpersonal communication about the benefits of OPV, assuage false fears such as the rumour that OPV causes sterility, and address other health grievances have dramatically reduced “resistance” to OPV [25], [31]. Furthermore, UNICEF's Underserved Strategy and Social Mobilization Network (SMNet), whom this study was conducted with, had improved communication between the GPEI and local communities by holding educational skits and plays about polio, and recruiting grassroots stake holders such as religious clerics to advocate for vaccination [32], [33].

The proposed goal of these IEC activities is to provide accurate information and correct misunderstandings [34]. The SMNet and Underserved Strategy have been very successful and accomplished this goal by reducing “resistance” across the board. As the roots of “resistance” change, it is necessary to modify what is targeted. During the time of this study, the “resistance” shifted from sterilization rumours to fatigue and fear about vaccine policy and safety. If this situation was to arise in India again, or currently stands in the other polio endemic nations, it may be necessary to provide accurate information about vaccine policy and safety, empowering the public to make the right decisions for the health of their children.

For example, if the public had been informed of the strategy to eradicate P1 first, they would have been able to follow case type, vaccinate against P3 if they wanted, and note the progress of the program. It could have been widely publicized that P1 cases fell by 51% from 2008–2009 to increase confidence in the program [35]. Even less educated families would probably have understood that there were different types of polio, and thus upon hearing of, witnessing, or as seen in this study, experiencing the occurrence of other cases of AFP, understand that it may not have been due to OPV efficacy alone. Some may contend that this would become a huge and difficult task. Indeed it may since health literacy about serotypes is low in developed countries as well. However, since only polio remains the principal disease of eradication in a pseudo “opt-in” format with massive campaigns at a national level, adding this level of detail need not be ruled out.

If the public had been involved from the beginning, it is possible that some of the resistance may have been reduced. Families were especially frustrated with the top-down nature of the polio program since it was not their principle priority. With open sewers, diarrheal illnesses, and unpaved roads: their priority was development. During this and previous studies [1], [2], some families agreed to vaccinate only if roads were built and other medical services provided. A large part of the Underserved Strategy with the SMNet had to be dedicated to building this bridge in the end. Though the idea of “disease eradication” garners more attention and drives international funding, pursuing eradication without communicating with the public may have simple dragged the program out longer then it needed to be.

Though the introduction and wide success of the bivalent OPV solved most of the problems discussed here the monovalent strategy, drastically reducing both P1 and P3 with about the same serconversion rates as the monovalent vaccines [36], it remains important to remember that the problems posed by lack of transparency likely persist and should be considered as policy shifts continue to occur.

An additional concern with the current strategy is that of medical ethics. Of the four medical principles, justice, beneficence, nonmaleficence, and autonomy, not informing the public of the small risks associated with OPV vaccination may impinge on the principle of autonomy: “giving patients the right to make their own choices” [37]. If patients are compelled to make a decision without access to information which could be provided, as seemed to be the case from the study where they were told that the vaccine is completely safe and has no side effects, it would prove problematic. The same issue would be the case with a dearth of information about the monovalent strategy.

To address this issue in future programs or countries still endemic with polio, increased transparency coupled with an adverse-effects compensation program could be considered. Many people had advocated for the introduction of inactivated polio vaccine (IPV) for eradication because it does not have the associated risk of VAPP like OPV [21], [38]. However, the cost and difficulty in administering IPV, which will not be discussed here, makes this problematic. Rather, by increasing transparency about the risks associated with OPV, the public could be empowered, restoring the principle of autonomy. As there should be few cases of VAPP and VDPV, especially with the success of the bivalent vaccine, the compensation program may may provide a more equitable alternative.

### Conclusions

A lack of transparency about the polio eradication program appeared to have contributed to “resistance” to vaccination in Aligarh in 2009. Families who had not been informed of the intensification of the program had come to doubt the vaccine's efficacy as polio cases occurred. This doubt seemed often exacerbated by the lack of transparency about the monovalent strategy to eradicate P1 as families had no way to differentiate polio serotypes. Many families in the study had even become fearful of the vaccine itself from what they perceived to be adverse events after being told there was no risk with vaccination. Though India has almost eradiated polio, the lessons learned here about the nature of social resistance should be considered to build and keep trust with the public in other polio-endemic regions and future eradication efforts.
